# Clinical trial: Chidamide plus CHOP improve the survival of newly diagnosed angioimmunoblastic T-cell lymphoma

**DOI:** 10.3389/fimmu.2024.1430648

**Published:** 2024-08-20

**Authors:** Xiangping Zong, Zhen Yang, Jin Zhou, Zhengming Jin, Depei Wu

**Affiliations:** ^1^ National Clinical Research Center for Hematologic Diseases, Jiangsu Institute of Hematology, The First Affiliated Hospital of Soochow University, Suzhou, China; ^2^ Institute of Blood and Marrow Transplantation, Collaborative Innovation Center of Hematology, Soochow University, Suzhou, China

**Keywords:** angioimmunoblastic T-cell lymphoma (AITL), chidamide, CHOP, transplantation, survival

## Abstract

**Background:**

Angioimmunoblastic T-cell lymphoma (AITL) is known for its unfavorable survival prognosis. Chidamide has shown efficacy in relapsed/refractory AITL, but its efficacy in newly diagnosed AITL is uncertain

**Objective:**

This retrospective research aimed to evaluate the effectiveness and safety of chidamide when used with doxorubicin, cyclophosphamide, prednisone, and vincristine (CHOP) in comparison to CHOP by itself for individuals newly diagnosed with AITL, and to examine the impact of transplantation.

**Method:**

This was an analysis that compared outcomes among patients who received chidamide + CHOP on a clinical trial vs. historical controls who received CHOP alone, enrolling a total of sixty-six treatment-naive AITL patients between April 2014 and November 2022. Among them, thirty-three received chidamide in addition to CHOP (chidamide group), while thirty-three received CHOP alone (control group). The clinical characteristics were balanced between the two groups. All patients were scheduled to undergo up to six courses of treatment before transplantation.

**Results:**

The chidamide group had a significantly longer median overall survival (OS) compared to the control group, with a median OS that was not reached, as opposed to 20 months in the control group (p = 0.002). In the control group, the median progression-free survival (PFS) was 11 months, while in the chidamide group, it was 22 months (p = 0.080). In the high-risk group (IPI ≥ 3), the chidamide group demonstrated notably superior complete response (CR) and overall response rate (ORR) compared to the control cohort (p = 0.002, p = 0.034). The PFS and OS in the chidamide group were not reached, and there were significant differences compared to the control group (p = 0.007, p = 0.003). The median OS of the transplanted group was longer than the non-transplanted group (p = 0.004). On multivariate analysis, chidamide group reduced the hazards of death in the total cohort.

**Conclusion:**

As the study was non-random and retrospective, Chidamide combined with chemotherapy should be tested in randomized trials given its potential to improve prognosis in treatment-naive AITL patients. Furthermore, autologous hematopoietic stem cell transplantation (auto-HSCT) has demonstrated enhanced overall survival in individuals with AITL.

**Clinical trial registration:**

https://clinicaltrials.gov/, NCT03268889

## Introduction

Angioimmunoblastic T-cell lymphoma (AITL) is an uncommon form of mature T-cell lymphoma, accounting for 1%-2% of non-Hodgkin lymphoma cases and 15%-20% of peripheral T-cell lymphoma (1). AITL is characterized by distinct clinical and pathological features, and it is associated with a poor prognosis (2, 3). The management of AITL remains challenging, as patients frequently experience relapse following initial and subsequent treatments (4). Currently, AITL is managed in a comparable manner to PTCL, not otherwise specified (PTCL, NOS), with the utilization of CHOP chemotherapy and auto-HSCT (5–7). Long-term results with CHOP have been explained by retrospective data, such as the International T-cell lymphoma project. In this study, 85% of patients received CHOP-based therapy, with AITL identified in 18.5% of them. AITL patients had a 5-year PFS rate of just 18% (8).

Histone deacetylase inhibitors and hypomethylating medications are examples of epigenetic modifiers that have selective activity in relapsed or refractory AITL (4). Chidamide, a novel histone deacetylase inhibitor, targets and blocks HDAC1, 2, 3, and 10 variants, leading to alterations in chromatin configuration and gene activity. This leads to the induction of tumor cell apoptosis and differentiation, inhibition of tumor angiogenesis and metastasis, and enhancement of the immune system’s killing effect on tumors (9).

Chidamide is a medication that was independently created and manufactured in China. The National Food and Drug Administration authorized it in 2015 as a treatment for relapsed or refractory PTCL. In clinical trials, chidamide monotherapy achieved an objective response rate (ORR) of 28%, a median PFS of 2.1 months, and a median OS of 21.4 months in relapsed or refractory PTCL. In AITL patients, higher ORR (50%) and complete response/unconfirmed complete response rates (40%) have been observed with chidamide treatment. Furthermore, the responses to chidamide treatment in AITL patients are longer-lasting (10). Chidamide maintenance therapy is also associated with improvements in OS and PFS in patients with PTCL who are not candidates for autologous stem cell transplantation while maintaining a manageable safety profile, and enrolled patient patients were required to have achieved a complete response (CR) or partial response (PR) after first-line regimen introduction (11).

The purpose of this article is to compare the effectiveness and safety of chidamide when used with the CHOP regimen versus using CHOP alone in newly diagnosed patients with AITL. Furthermore, the research seeks to assess the impact of transplantation in AITL. We believe that this clinical study is of great value and will contribute to expanding treatment options for AITL patients.

## Materials and methods

### Study design and participants

The study that compared outcomes among patients who received chidamide + CHOP on a clinical trial vs. historical controls who received CHOP alone was carried out to assess the efficacy and safety of CHOP therapy, with or without chidamide, in a cohort of 66 newly diagnosed AITL patients, ranging in age from 25 to 86 years. The study took place at the First Affiliated Hospital of Soochow University from April 2014 to November 2022. The chidamide cohort comprised patients who engaged in a clinical investigation identified as NCT03268889; conversely, the control cohort consisted of subjects who had received the CHOP regimen at our institution prior to the commencement of the trial. The research was carried out following ethical guidelines and the standards outlined in the Helsinki Declaration. Our center’s ethical committee had approved the protocol, informed consent form, and other necessary study materials. Patients in the Chidamide group submitted written informed consent before participation in the study.

Participants in this research were required to satisfy the following conditions: (1) AITL pathology diagnosis based on the 2016 WHO classification (12), validated by a central review involving hematopathological specialists; (2) over 18 years old; (3) Eastern Cooperative Oncology Group score ranging from 0 to 3, with a projected survival exceeding 3 months (patients with a score of 3 had high tumor load, and all patients in the article were able to tolerate chemotherapy). Laboratory findings were considered satisfactory if the peripheral blood neutrophil count was at least 1.5×109/L, platelet count was at least 20 ×109/L, hemoglobin was at least 70 g/L, and renal function was intact with a creatinine clearance rate of no less than 30 mL/min, while alanine aminotransferase (ALT) and aspartate aminotransferase (AST) levels remained within three times the upper limit of the normal range.

Patients with central nervous system involvement, severe acute infection, or major heart disease were excluded.

### Treatment regimens

All 66 patients were newly diagnosed with AITL. 33 patients were assigned to the chidamide plus CHOP group, with another 33 patients in the control group receiving only the CHOP chemotherapy regimen.

Patients in the chidamide plus CHOP cohort were administered up to 6 cycles of treatment, which comprised cyclophosphamide administered at a dose of 750 mg/m2 on the initial day, followed by doxorubicin at 50 mg/m2 on the same day. Vincristine was given at a dosage of 1.4 mg/m2 on day 1, and oral prednisone was taken daily, starting from day 1 and continuing through day 5, with a dose of 100 mg each day. The process was repeated every three weeks, with the addition of oral chidamide (30mg) twice weekly. The control group received the CHOP treatment. Upon finishing the initial 3 rounds, the treatment’s efficacy was assessed with PET-CT, in accordance with the latest evaluation standards for non-Hodgkin’s lymphoma. Patients with progressive disease (PD) or stable disease (SD) were excluded from the study and received second-line therapy. Patients who achieved either CR or PR were given three additional cycles to complete the planned six cycles, and their effectiveness was reassessed using PET-CT. Subsequent treatment options included the choice to undergo autologous stem cell transplantation consolidation treatment.

Dose adjustments were contingent upon the intensity and category of adverse effects. A reduction to one-third of the CHOP dosage was necessitated by hematological events, specifically grade-3 thrombocytopenia or grade-4 neutropenia occurring from day 1 through day 21. No provisions were made for altering chidamide doses. For non-hematological toxicities, the decision to decrease the dose was on the treating physicians’ judgment.

### Study endpoint

The primary endpoint was to compare the observation group and the control group, respectively in the overall and high-risk group (IPI ≥ 3) of the ORR, CR, 2-year OS, 2-year PFS, and adverse events (AEs) of the two groups. At the same time, the PFS and OS of the transplant group and the non-transplant group were observed. The evaluation of AEs was conducted in accordance with the National Cancer Institute’s Common Terminology Criteria for Adverse Events, version 5.0.

### Statistical analysis

Continuous variables were presented as medians and ranges, whereas categorical variables were represented as frequencies and percentages. Continuous variables were compared using the Mann-Whitney U test. The ORR and CR rates were compared using the cross-tabulation χ2 test. PFS and OS were estimated using the Kaplan–Meier method and log-rank test. Patients who were lost to follow-up were censored on their final follow-up date. A Cox proportional hazards regression model was employed to explore the variables affecting survival, conducting both Univariate Analysis (UVA) and Multivariate Analysis (MVA). A two-tailed test with a P-value threshold of less than 0.05 was adopted to establish statistical significance. All the analytical procedures were carried out using SPSS software, version 27.0 (by IBM Corp., located in Chicago, IL), along with GraphPad Prism version 9.5.

## Results

### Patient characteristics

This study included 66 patients diagnosed with AITL, divided into two groups: 33 patients received chidamide in combination with CHOP, while the remaining 33 patients were in the control group. **Table 1** shows that the baseline characteristics of both groups were similar with no notable distinctions. In the entire group of patients, the middle age was 62 years (ranging from 25 to 86 years), with 47 male patients making up 71.2%. 61 patients (92.4%) had Ann Arbor stage III or above. Among the patients, 23 had B symptoms (34.8%), 24 had serous effusion (36.3%), 20 had EBV viremia (30.3%), and 9 had hemolytic anemia (13.6%). Additionally, 36 patients had an International Prognostic Index (IPI) score of ≥ 3 points, accounting for 54.5% of the cohort.

**Table 1:** Baseline characteristics of 66 AITL patients [n (%)].

Characteristics	Control group (n=33)	Chidamide group (n=33)	P
Male	22 (66.7)	25 (75.8)	0.415
Age, years, median (range)	59 (35.0-86.0)	64 (25-79)	0.476
WBC (<4×10^9^/L)	8 (24.2)	7 (21.2)	0.769
HB (<100 g/l)	10 (30.3)	8 (24.2)	0.580
PLT (<100×10^9^/L)	8 (24.2)	6 (18.2)	0.547
ALB (<35 g/l)	20 (60.6)	15 (45.5)	0.218
β2-MG >ULN	15 (45.5)	12 (36.4)	0.453
LDH >ULN	17 (51.5)	17 (51.5)	1.000
Extranodal sites ≥2	13 (39.3)	11 (33.3)	0.609
EBV viremia	9 (27.3)	11 (33.3)	0.592
Hemolytic anemia	5 (15.2)	4 (12.1)	1.000
Bone marrow involvement	7 (21.2)	6 (18.2)	0.757
Serous effusion	14 (42.4)	10 (30.3)	0.306
B symptoms	11 (33.3)	12 (36.4)	0.796
Stage III-IV	30 (90.9)	31 (93.9)	1.000
IPI≥3	16 (48.5)	20 (60.6)	0.323
HSCT	7 (21.2)	12 (36.4)	0.174

AITL, angioimmunoblastic T-cell lymphoma; WBC, white blood cell; HB, haemoglobin; PLT, platelet; ALB, albumin; ULN, upper limit of normal; LDH, lactic dehydrogenase; EBV, Epstein-Barr virus; IPI, International Prognostic Index; HSCT, hematopoietic stem cell transplantation.

**Figure 1:**
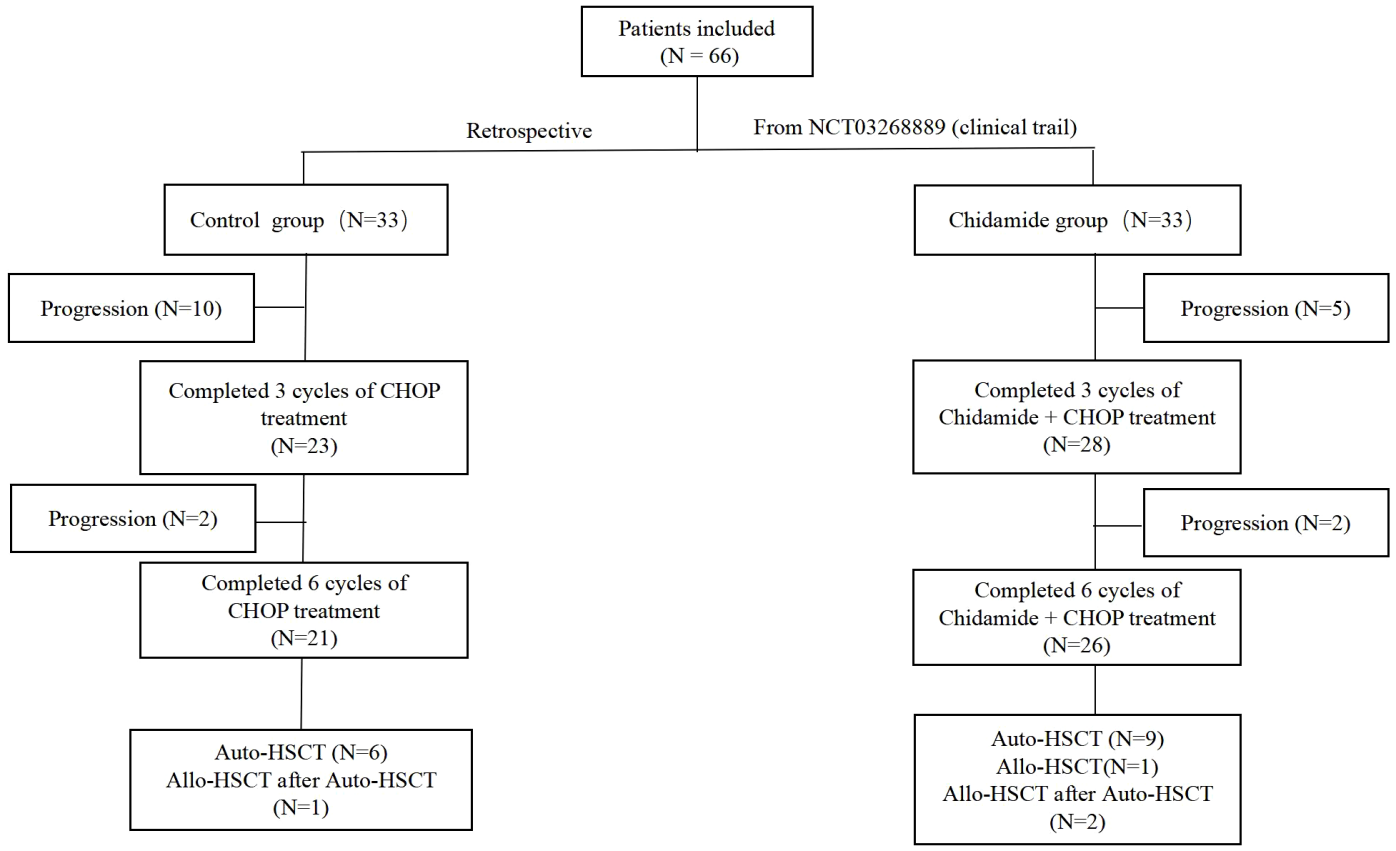
Schema of patients included in analysis.

In the control group, 21 patients completed 6 cycles of CHOP chemotherapy, 12 patients experienced disease progression during the treatment, and 5 patients died while undergoing treatment. In the chidamide group, 26 patients completed 6 cycles of chidamide combined with CHOP chemotherapy, 7 patients experienced disease progression, and 3 patients died during the treatment. A total of 18 patients underwent autologous hematopoietic stem cell transplantation (11 in the chidamide group), and 3 patients underwent allogeneic hematopoietic stem cell transplantation (allo-HSCT) after the failure of autologous hematopoietic stem cell transplantation (auto-HSCT) (2 in the chidamide group). In the chidamide group, one patient underwent allo-HSCT directly due to failure of autologous stem cell mobilization (**Figure 1**).

### Treatment regimens and outcomes

66 patients completed the treatment and were evaluated, with a median follow-up time of 15 months. The predicted median OS was 65 months (95% CI 7–122 months), and the 2-year and 5-year OS rates were 55% and 51.4%, respectively. The median PFS was 16 months (95% CI 10–21 months), and the 2-year and 5-year PFS rates were 39.3% and 32.9%, respectively. In the control group, 14 patients achieved CR (42.4%) after chemotherapy, while 24 patients (72.7%) achieved CR in the chidamide group (**Table 2**). The predicted median OS in the control group was 20 months (95% CI 14.1–25.8 months), whereas the OS in the chidamide group was not reached (P = 0.002). The median PFS in the control group was 11 months (95% CI 6.1–15.8 months) while the PFS in the chidamide group was 22 months (95% CI 10.5–33.5 months) (P = 0.086). In the control cohort, the 2-year overall survival (OS) and progression-free survival (PFS) rates stood at 36.7% and 35.4%, respectively, whereas the 5-year OS and PFS were calculated to be 15.3% and 14.1%. On the other hand, the group receiving chidamide demonstrated a 2-year OS and PFS of 79.2% and 43.6%, translating to 5-year OS and PFS rates of 72.6% and 38.1%. The mean time until recurrence or progression of all was 12.3 months, with the chidamide group registering 14 months compared to 9.7 months in the control group.

**Table 2:** The therapeutic efficacy between the two groups and IPI≥3 [n (%)].

	Control group (n=33)	Chidamide group (n=33)	P
CR	14 (42.4)	24 (72.7)	0.013
ORR	21 (63.6)	27 (81.8)	0.097
Relapse and progress	21(63.6)	16 (48.5)	0.215
PFS (M, 95%CI)	11 (6.1-15.8)	22 (10.5-33.5)	0.080
OS (M, 95%CI)	20 (14.1-25.8)	–	0.002
IPI≥3	Control group (n=16)	Chidamide group (n=20)	
CR	3 (18.7)	15 (75.0)	0.002
ORR	8 (50.0)	17 (85.0)	0.034
Relapse and progress	14 (87.5)	9 (45.0)	0.014
PFS (M, 95%CI)	8 (2.1-13.8)	24	0.007
OS (M, 95%CI)	14 (7.4-20.5)	–	0.003

CR, complete response; ORR, overall response rate; PFS, progression-free survival; M, month; CI, confidence interval; OS, overall survival; IPI, International Prognostic Index; -, not reached.

**Figure 2:**
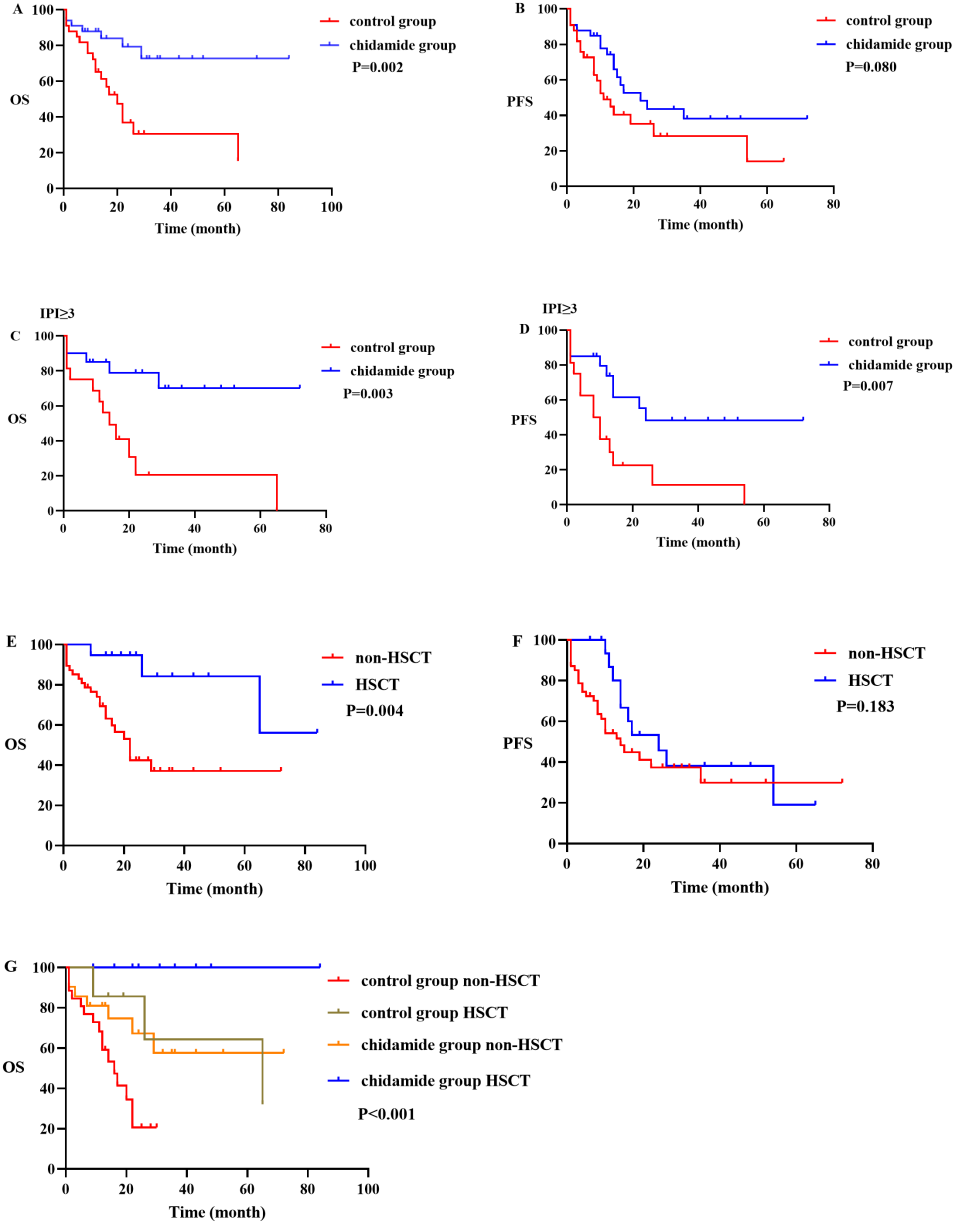
OS and PFS between different subgroups. **(A, B)** graphs showing OS and PFS in control group and chidamide group; **(C, D)** graphs showing OS and PFS in IPI ≥ 3 of the control group and chidamide group; **(E, F)** graphs showing OS and PFS in the non-HSCT group and HSCT group; **(G)** graph showing OS in the control group and chidamide group of HSCT and non-HSCT.

In the high-risk group with an IPI ≥ 3, there were 16 cases in the control group and 20 cases in the chidamide group. The chidamide group had a significantly higher CR rate and ORR than the control group (P = 0.002, P = 0.034), as well as a significantly reduced relapse or advancement rate (50% vs.85%, P = 0.014). In the high-risk group, the chidamide group had longer OS and PFS rates than the control group (P = 0.003, P = 0.007) (**Figure 2**). However no differences were referring to OS and PFS in IPI<3 of the control group and Chidamide group (P = 0.234, P = 0.168).

### Survival after transplantation

A total of nineteen patients underwent hematopoietic stem cell transplantation (HSCT), and nine of them experienced a recurrence after undergoing autologous HSCT. In addition, 47 patients did not undergo transplantation, and out of these, 27 experienced relapse or disease progression. At the time of follow-up, 25 patients had died. The two-year OS and PFS rates for the transplanted patients were 94.7% and 45.7%, respectively. The five-year OS and PFS rates were 84.2% and 19%, respectively. As for the non-transplanted patients, their two-year OS and PFS rates were 42.4% and 37.4%, respectively, while their five-year OS and PFS rates were 37.1% and 29.9%, respectively. The median OS for the non-transplanted group was 22 months, whereas the median OS for the transplanted group was not reached. A statistically significant distinction was observed between the two groups (p = 0.004) (**Figure 2**). In terms of PFS, the non-transplanted group had a median PFS of 14 months, compared to 24 months for the transplanted group. The difference in PFS between the two groups was not statistically significant (p = 0.183). Within this study, there were a total of 6 cases that were in either PR or NR status before transplantation. These patients underwent auto-HSCT and had a median remission time of 5 months (range 2–8 months). All of them experienced relapse, and out of these, 3 underwent allo-HSCT again and achieved sustained remission. In terms of allo-HSCT, all 4 patients who underwent the procedure survived.

There were 26 patients in the control group with non-HSCT and 7 patients in the control group with HSCT, while 21 patients in chidamide group with non-HSCT and 12 patients in chidamide group with HSCT. The OS among the four above groups were significantly different (p <0.001) (**Figure 2**). Patients in the chidamide group without HSCT had a longer OS than patients in the control group without HSCT (p = 0.022). However, the difference in OS between patients in the chidamide group with HSCT and the control group with HSCT was not so obvious (p = 0.070). The OS with HSCT were longer than that without HSCT in chidamide group and control group respectively, (p = 0.044, p = 0.031). Interestingly, there was no difference in OS between the chidamide group without HSCT and the control group with HSCT (p=0.884) (**Supplementary Table 1**).

### Assessment of factors affecting survival

Cox regression analysis was utilized to identify the factors that predict OS, as shown in **Table 3**. We included Chidamide group, age ≥ 60 years, IPI ≥ 3, and HSCT or not as variables. Chidamide group and HSCT on UVA showed reduced risks of mortality, while age ≥ 60 years and IPI ≥ 3 had no impact on mortality risk. On MVA, the same attributes were maintained. Chidamide group and HSCT reduced the hazard of death on both UVA and MVA. The hazard was not affected by the evaluation of the same variables for assessing PFS (**Supplementary Table 2**).

**Table 3:** Univariate and multivariable analysis of factors affecting OS of AITL.

OS							
UVA				MVA			
Variable	HR	95%CI	P	Variable	HR	95%CI	P
Chidamide group	0.27	0.11-0.66	0.004	Chidamide group	0.27	0.11-0.67	0.005
Age ≥60 years	0.90	0.41-1.96	0.794				
B symptoms	1.37	0.64-2.98	0.416				
Stage III-IV	22.59	0.02-26542.54	0.387				
Bone marrow involvement	1.06	0.42-2.67	0.896				
Extranodal sites ≥2	1.51	0.70-3.29	0.293				
IPI≥3	1.56	0.69-3.51	0.280				
HSCT	0.20	0.05-0.68	0.010	HSCT	0.19	0.05-0.69	0.012

OS, overall-survival; HR, Hazard ratio; UVA, Univariate Analysis; MVA, Multivariate Analysis; IPI, International Prognostic Index; HSCT, hematopoietic stem cell transplantation; CI, Confidence Interval. Only factors significant on UVA at P < 0.05 or relevant to the prognosis were used in MVA.

### Toxicity profiles

Hematological adverse events were the most commonly reported treatment-related side effects. **Table 4** provides a summary of the adverse events reported in both groups, including hematological adverse events, liver function impairment, renal impairment, nausea/vomiting, and rash. In the control cohort, 51.5% experienced grade 3/4 neutropenia, compared to 57.6% in the chidamide cohort. Grade 3/4 anemia occurred in 12.1% and 15.2% of patients in these groups, correspondingly. Likewise, the incidences of grade 3/4 thrombocytopenia stood at 12.1% and 21.2%. Both regimens primarily led to grade nausea/vomiting and fatigue as frequent non-hematological side effects. No statistically noteworthy disparity was found in the frequency of adverse events between the therapy arms. Importantly, no treatment-related fatalities were documented.

**Table 4:** All grade toxic events [n (%)].

	All Grade		Grade>=3	
	Control group (n=33)	Chidamide group (n=33)	Control group (n=33)	Chidamide group (n=33)
Hematological				
Neutropenia	21 (63.6)	23 (69.7)	17 (51.5)	19 (57.6)
Anemia	17 (51.5)	26 (78.8)	4 (12.1)	5 (15.2)
Thrombocytopenia	20 (60.6)	20 (60.6)	4 (12.1)	7 (21.2)
Non-hematological				
ALT or AST elevation	1 (3.0)	0	0	0
Elevated creatinine	1 (3.0)	0	0	0
Hyperuricemia	1 (3.0)	1 (3.0)	0	0
Pneumonia	1 (3.0)	1 (3.0)	0	0
Nausea/vomiting	4 (12.1)	4 (12.1)	2 (6.1)	2 (6.1)
Fatigue	9 (27.3)	10 (30.3)	0	0
Hyponatremia	8 (24.2)	10 (30.3)	0	1 (3.0)
Rash	0	3 (9.1)	0	0

ALT, alanine aminotransferase; AST, aspartate aminotransferase.

## Discussion

This study observed that the first-line use of chidamide combined with CHOP in newly diagnosed AITL patients resulted in longer OS compared to the CHOP group (p = 0.002). In patients at high risk with an IPI score of 3 or higher, there was a significant improvement in CR, PFS, and OS (p = 0.002, p = 0.007, p = 0.003, respectively). Moreover, hematopoietic stem cell transplant has been shown to enhance patient survival, particularly in those who did not reach complete remission, with allogeneic transplant demonstrating encouraging outcomes. Hematological toxicities were the most frequent adverse events associated with treatment, and no notable distinction was found between the two groups. The results indicate that chidamide in combination with CHOP can be a viable and well-tolerated treatment choice for AITL patients who have not received treatment before, particularly those in the high-risk category. The addition of Romidepsin to CHOP did not result in improved progression-free survival in untreated PTCL patients, but it did demonstrate effectiveness in the TFH subtype (6), aligning with our research findings. Our study first revealed the efficacy of chidamide in conjunction with CHOP in AITL, and then compared it retrospectively to the control group of CHOP alone, finding the superiority of chidamide in first-line treatment of the AITL subtype. AITL mainly originates from TFH cells and has unique biological characteristics and clinical manifestations (3).

AITL has a distinct gene expression profile and multi-step pathogenesis, characterized by epigenetic abnormalities at the hematopoietic stem cell stage, primarily involving TET2, DNMT3A, and IDH2 mutations. During the mature T cell phase, the emergence of tumor-specific mutations like RHOA (G17V) and IDH2 (R172) play a role in the progression of AITL (13, 14). Chidamide, an orally available HDAC inhibitor, targets class I HDAC1/2/3 and class IIb HDAC10 of histone deacetylases. It can inhibit the growth and metastasis of PTCL by regulating epigenetic changes (15). Romidepsin is also one of the histone deacetylase inhibitors. Oral 5-azacytidine and romidepsin show significant efficacy in individuals diagnosed with PTCL (16).

Furthermore, a study in Japan examined the effectiveness of chidamide as a standalone treatment for 55 patients with relapsed or refractory PTCL (17). The research indicated a 46% ORR and a median OS of 23 months. A different research conducted by Sun Yat-sen University Cancer Center investigated how effective chidamide, when combined with a chemotherapy plan, could be as the initial treatment for 104 PTCL patients (including PTCL-NOS, AITL and others). It was a single-arm prospective study combined with historical control study. The research indicated that the chidamide combination resulted in a higher ORR of 77.4% compared to 72.6% with chemotherapy alone, along with a longer median PFS of 12.4 months versus 8.5 months (p = 0.047) (18). In our study, we observed a CR rate of 72.7% versus 42.4% (p = 0.013), a median PFS of 22 months versus 11 months (p = 0.080), OS of not reached versus 20 months (p = 0.002) in the chidamide group compared to the control group. Compared with the chidamide group, twice as many people in the historical control group progressed after the first 3 cycles of treatment. These were in line with previous reports showing a benefit of histone deacetylase inhibitors in PTCL exhibiting a TFH phenotype (6). Our study found that, in high-risk AITL patients with an IPI ≥ 3, the chidamide group combined with chemotherapy had significantly higher CR and ORR rates compared to the control group (75% vs. 18.7%, p = 0.002, 85% vs.50%, p = 0.034). Additionally, PFS and OS also showed significant benefits (p = 0.007, p = 0.003). We also verified that chidamide was an independent protective factor in reducing mortality through Cox regression analysis (HR, 0.27; 95% CI, 0.11–0.67; p = 0.005). Based on the data from the beforementioned clinical studies, it can be inferred that AITL may benefit more from chidamide treatment than PTCL. This finding is also associated with the epigenetic abnormalities underlying the pathogenesis of AITL.

The efficacy of ASCT as a primary treatment remains a topic of debate. The Nordic Lymphoma Group conducted a significant prospective study, which observed extended PFS, with a median duration exceeding 30 months, following six rounds of CHOEP therapy (CHOP plus etoposide) and subsequent ASCT as part of the conditioning regimen (19). However, in the absence of a randomized control group not undergoing ASCT, drawing conclusive evidence remains challenging. A multicenter retrospective analysis upheld the substantial improvement in outcome for AITL patients who received autologous stem cell transplantation subsequent to high-dose chemotherapy. The investigation revealed that individuals who underwent transplantation during their first complete remission exhibited superior long-term survival odds compared to those who had rescue transplantation, with a 4-year progression-free survival rate of 56%. In contrast, relapsed and refractory AITL patients had PFS rates of 30% and 23%, respectively (20). In our study, we discovered that the 5-year OS rates of patients with AITL who underwent transplantation and those who did not were 84.2% and 37.1%, respectively (p = 0.004).

In this study, whether the patient underwent HSCT was comprehensively evaluated according to whether the patient was sensitive to the previous chemotherapy, the patient’s physical condition, economic status and willingness. On UVA and MVA, HSCT was found to be a protective factor of OS. In a separate retrospective study, it was found that among patients who relapsed after achieving CR for the first time and underwent re-treatment to attain CR or PR, allogeneic transplantation demonstrated improved OS (4-year OS, 80%; 95% CI, 55–92%) compared to autologous transplantation (4-year OS, 47%; 95% CI, 25–66%) (p = 0.043) (21). A total of 18 patients underwent auto-HSCT, of whom nine experienced relapse. Remarkably, six of these relapsed patients had not achieved CR status prior to transplantation. Among them, three underwent allogeneic transplantation and achieved sustained remission. It is worth highlighting that no transplant-related mortalities were recorded. These findings reinforce the notion that the survival outcomes following allo-HSCT for relapsed or treatment-resistant PTCL-NOS or AITL are comparable to those of auto-HSCT.

This study has several limitations. First, the follow-up time of the study was relatively short. Longer follow-up on clinical outcomes is needed. Second, due to the small sample size of this study, patients were not stratified according to whether they were sensitive to previous chemotherapy and then divided into hematopoietic stem cell transplantation groups to assess survival time. Third, since the decision to perform prior autologous stem cell transplantation for these patients was made by the attending physician, there may be a potential for selection bias. Despite these limitations, the study was a retrospective cohort study that was able to assess the real-world situation of contemporary treatment approaches and patient outcomes. However, further data from prospective clinical studies are needed to determine the optimal type of transplantation.

## Conclusion

In summary, our findings indicate that the incorporation of chidamide into chemotherapy for AITL patients can improve the remission rate and survival time, the efficacy should be tested in randomized trials. Additionally, transplantation has shown promise in enhancing patient survival time.

## Data Availability

The original contributions presented in the study are included in the article/**Supplementary Material**. Further inquiries can be directed to the corresponding authors.
