# Supplementary material for: Clinical trial: Chidamide plus CHOP improve the survival of newly diagnosed angioimmunoblastic T-cell lymphoma
Source: Front Immunol. 2024 Aug 20;15:1430648. doi: 10.3389/fimmu.2024.1430648 (PMC11368836; doi:10.3389/fimmu.2024.1430648)
Supplement: Supplementary file 1 [file Table1.docx]

Supplement table 1. OS compared between different subgroups.

| OS (M, 95%CI) | | VS | OS (M, 95%CI) | | P |
| --- | --- | --- | --- | --- | --- |
| non-HSCT n=47 | | | HSCT n=19 | |  |
| 22( 16.0-27.0) | | | - |  | 0.004 |
| non-HSCT (control group) n=26 | | | HSCT (control group) n=7 | | |
| 16 (9.9-22.0) | | | 65 (5.4-124.5) | | 0.044 |
| non-HSCT (chidamide group) n=21 | | | HSCT (chidamide group) n=12 | | |
| - |  | | - |  | 0.031 |
| non-HSCT (control group) n=26 | | | non-HSCT (chidamide group) n=21 | | |
| 16 (9.9-22.1) | | | - |  | 0.022 |
| HSCT (control group) n=7 | | | non-HSCT (chidamide group) n=21 | | |
| 65 (5.4-124.5） | | | - |  | 0.884 |
| HSCT (control group) n=7 | | | HSCT (chidamide group) n=12 | | |
| 65 (5.4-124.5） | | | - |  | 0.070 |

# Notes: OS: overall survival; M: month; CI: confidence interval; -: not reached; HSCT: hematopoietic stem cell transplantation, non-HSCT: not hematopoietic stem cell transplantation.
