# Supplementary material for: Clinical trial: Chidamide plus CHOP improve the survival of newly diagnosed angioimmunoblastic T-cell lymphoma
Source: Front Immunol. 2024 Aug 20;15:1430648. doi: 10.3389/fimmu.2024.1430648 (PMC11368836; doi:10.3389/fimmu.2024.1430648)
Supplement: Supplementary file 2 [file Table2.docx]

Supplemental Table 2. Univariate analysis of factors affecting PFS of AITL.

| PFS | | | |
| --- | --- | --- | --- |
| Variable | HR | 95%CI | P |
| Chidamide group | 0.57 | 0.29-1.10 | 0.095 |
| Age ≥60 years | 0.81 | 0.42-1.55 | 0.539 |
| IPI≥3 | 1.30 | 0.66-2.53 | 0.440 |
| HSCT | 0.61 | 0.29-1.28 | 0.195 |

Notes: PFS: progression-free survival; AITL: Angioimmunoblastic T-cell lymphoma; HR: Hazard ratio; CI: Confidence Interval; IPI: International Prognostic Index; HSCT: hematopoietic stem cell transplantation.
